# Incidence of asymptomatic neurosyphilis in serofast Chinese syphilis patients

**DOI:** 10.1038/s41598-017-15641-w

**Published:** 2017-11-13

**Authors:** Sheng Nan Cai, Jing Long, Chen Chen, Gang Wan, Wen Hui Lun

**Affiliations:** 1 Peking University Ditan Teaching Hospital, Beijing, China; 2 Beijing Ditan Hospital, Capital Medical University, Beijing, China

## Abstract

More new diagnosed syphilis cases were reported in china, the incidence and relevant factors of asymptomatic neurosyphilis (ANS) in serofast syphilis patients were unclear. Clinical and laboratory data of 402 Human Immunodeficiency Virus (HIV) negative, serofast syphilis patients, who underwent lumbar puncture at the Peking University Ditan Teaching Hospital between September 2008 and August 2016, were collected. Incidence of ANS was verified and the relevant factors were further analyzed. According to the ANS criteria, 139 (34.6%) patients had ANS. Of these, 40 (28.8%) had reactive cerebrospinal fluid (CSF), rapid plasma reagin (RPR) positive, 115 (82.7%) had CSF white blood cell (WBC) count > 5 × 10^6^/L, 28 (20.1%) had CSF protein concentration > 45 mg/dL (without other neurological diseases). Patients aged 51–60 years, of non-Han ethnicity, with serum RPR titer 1:32 and ≥ 1:64 were 2.28-fold, 9.11-fold, 5.12-fold and 5.69-fold, respectively, more likely to have ANS. The incidence of ANS was 34.6% among Chinese serofast syphilis patients. Age, ethnicity and serum RPR titer were associated with high risk of ANS.

## Introduction

The incidence of syphilis is rapidly increasing in China^1^. Syphilis is a systemic and infectious disease caused by Treponema pallidum, which can disseminate to any organ shortly after infection. Neurosyphilis can occur at any stage of syphilis, after invasion of the central nervous system by Treponema pallidum, and its most common form is asymptomatic neurosyphilis (ANS)^2^. ANS shows one or more CSF abnormalities (pleocytosis, or elevated protein concentration, or reactive cerebrospinal fluid (CSF) Venereal Disease Research Laboratory (VDRL) in patients with serological evidence of syphilis but no neurological signs or symptoms^3^. According to the World Health Organization (WHO), an estimated 12 million new cases of syphilis occur each year^4^, The incidence of neurosyphilis is also increasing, with an annual incidence of 0.16–2.1 per 100,000 population^5,6^. About 13.5% of latent syphilis patients without neurological symptoms had ANS, and these patients were more likely to have late neurological complications^7^. About 35% ANS patients developed symptomatic neurosyphilis^8^. In China, the incidence of serofast state was 34.4%, but the rate of ANS in the asymptomatic serofast syphilis patients remains unknown^9,10^. This study explored the incidence of ANS and relevant factors through analysis of clinical and laboratory data of 402 asymptomatic serofast syphilis patients.

## Results

### Characteristics of study patients

A total of 402 eligible patients were enrolled in the study. There were 142 and 260 male and female patients, respectively. Most patients were of Han ethnicity (293). The median age was 33 years (range: 17–79 years). A total of 139 (34.6%) patients were diagnosed with ANS according to the above criteria. Of these, 40 had reactive CSF RPR titer, of which 26 had RPR 1:1; nine had RPR 1:2, three had RPR 1:4; and two had RPR 1:8. A total of 115 patients had CSF WBC > 5 × 10^6^/L, the median was 10 × 10^6^/L (range: 0–216 × 10^6^/L); 28 patients had CSF protein concentration > 45 mg/dL, the median was 29.9 mg/dL (range: 7.2–216 mg/dL). Pretreatment serum RPR titer, serum RPR titer (when lumbar puncture was performed), fold decline of serum RPR titer after initial treatment, therapeutic regimen time between treatment and lumbar puncture and other detailed information are shown in Table 1.

**Table 1:** Patient Demographics and Baseline Characteristics (N = 402).

Variables (Units)	ANS (n = 139)	Serofast (n = 263)
Sex		
Male (n, %)	56(40.3)	86(32.7)
Female (n, %)	83(59.7)	177(67.3)
Age, (yrs, %)		
≤30	47(33.8)	115(43.7)
31–40	42(30.2)	78(29.7)
41–50	7(12.2)	31(11.7)
51–60	22(15.8)	24(9.1)
≥61	11(7.9)	15(5.7)
Ethnic (n, %)		
Han	132(95.0)	261(99.2)
Others	7(0.5)	2(0.8)
Marital status (n, %)		
Married	104(95.0)	189(71.9)
Single	28(20.1)	61(23.2)
Divorced	5(3.6)	9(3.4)
Widowed	1(0.7)	1(0.4)
Unknown	1(0.7)	3(1.1)
Pretreatment serum RPR titer (n, %)		
≤1:2	3(2.2)	16(6.1)
1:4	7(5.0)	24(9.1)
1:8	17(12.2)	40(15.2)
1:16	22(15.8)	45(67.2)
1:32	26(18.7)	41(17.1)
1:64	24(17.3)	28(10.6)
1:128	14(10.1)	20(7.6)
≥1:256	4(2.9)	8(3.0)
Unknown	22(15.8)	41(15.6)
Current serum RPR titer (n, %)		
1:1	4(2.9)	19(7.2)
1:2	13(9.35)	45(17.1)
1:4	20(14.4)	52(19.8)
1:8	33(23.7)	65(24.7)
1:16	24(17.3)	50(19.0)
1:32	28(20.1)	21(8.0)
≥1:64	17(12.2)	11(4.2)
Decline fold of serum RPR titer after initial treatment (n, %)		
<4-fold	74(53.2)	123(46.8)
≥4-fold	43(30.9)	99(37.6)
Unknown	22(15.8)	41(15.6)
Therapeutic regimen (n, %)		
Benzathine	129(92.8)	241(91.6)
Ceftriaxone	8(5.8)	12(4.6)
Tetracycline	1(0.7)	9(3.4)
Erythrocin	1(0.7)	1(0.4)
Duration between syphilis diagnose and lumber puncture (n, %)		
<12 m	21(15.1)	33(12.5)
≥12 m and <18 m	43(30.9)	77(29.3)
≥18 m and <24 m	14(10.1)	23(8.7)
≥24 m	61(43.9)	130(49.4)

Abbreviation: ANS = asymptomatic neurosyphilis; RPR = rapid plasma regain.

Some ANS patients met two or three diagnostic criteria. A total of 25 patients had both reactive CSF RPR titer and CSF WBC > 5 × 10^6^/L, nine patients had both reactive CSF RPR titer and CSF protein concentration > 45 mg/dL; 17 patients had both CSF WBC > 5 × 10^6^/L and CSF protein concentration >45 mg/dL and seven patients had CSF WBC > 5 × 10^6^/L, CSF protein > 45 mg/dL and the CSF RPR titer was reactive. 82.7% of ANS patients had CSF WBC > 5 × 10^6^/L, higher than those of reactive CSF-RPR (28.8%) and high CSF protein concentration (20.1%); the rate of ANS with at least two CSF abnormalities was 26.6%, and the rate of ANS with three abnormalities was 5.0% (Table 2).

**Table 2:** CSF characteristics of ANS patients.

CSF characteristics	CSF RPR (+) N(%)	CSF RPR (−) N(%)	
Reactive RPR titer			
WBC > 5 × 10^6^/L and protein > 45 mg/dL	7(5.0)	10(7.2)	
WBC > 5 × 10^6^/L and protein ≤ 45 mg/dL	18(12.9)	80(57.6)	
protein > 45 mg/dL and WBC ≤ 5 × 10^6^/L	2(1.4)	9(6.5)	
protein ≤ 45 mg/dLand WBC ≤ 5 × 10^6^/L	13(9.4)	—	
Total	40(28.7)	99(71.3)	139(100)

Abbreviation: CSF = cerebrospinal fluid; ANS = asymptomatic neurosyphilis; RPR = rapid plasma reagin; WBC = white blood cell.

### Relationship between serum RPR titer and ANS in asymptomatic serofast syphilis patients

**Figure 1:**
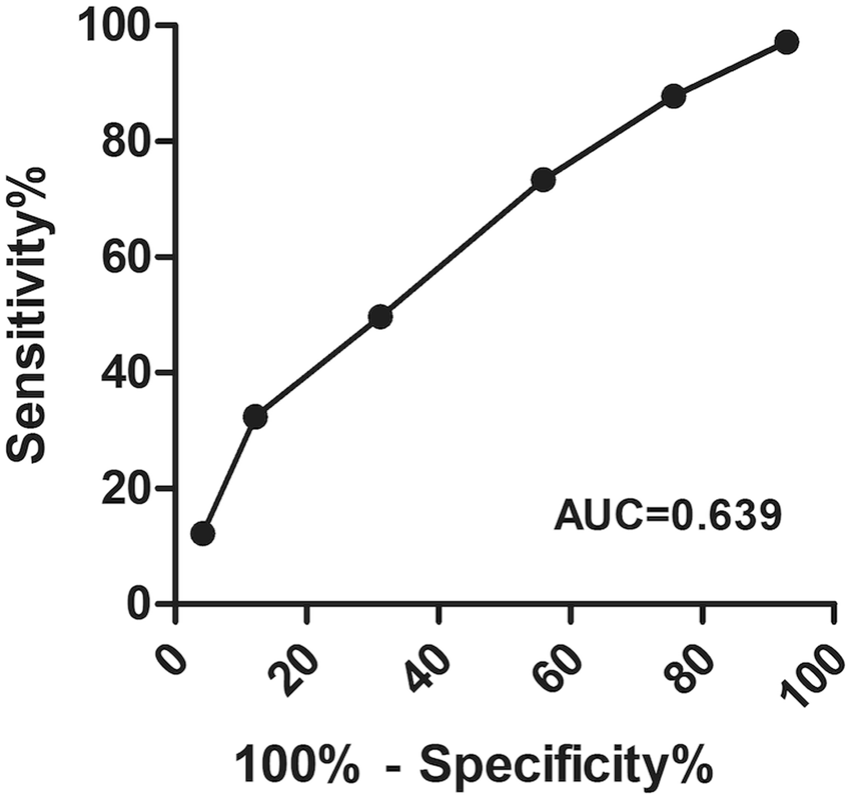
Receiver operating characteristic curve using serum RPR titer to distinguish ANS from asymptomatic serofast syphilis.

The proportions of ANS in asymptomatic serofast syphilis patients increased with the serum RPR titers (Table 1), the proportion of ANS ranged from 17.4% in the patients with RPR titers 1:1 to 60.7% in the patients with RPR titers ≥ 1:64. From the ROC curve, serum RPR titer 1:16 with the highest Youden index was the best cutoff (sensitivity 32.4%, specificity 87.8%). The area under the curve (95% CI) was 0.639 (0.610–0.668) (Fig. 1).

After the initial recommended treatment, 142 patients had a ≥4-fold decline of serum titer, of which 30.3% had ANS; 197 patients had a <4-fold decline of serum titer, and the proportion of ANS was 37.6%, which was higher than 30.3% but not statistically significant (p > 0.05). Hence, a 4-fold decline of serum titer after recommended treatment had no apparent influence on ANS, and could not be used to predict its occurrence.

### Relevant factors of asymptomatic neurosyphilis

The incidence of neurosyphilis and related factors has been extensively studied, but the literature on ANS is very limited. Our study explored the association of the above-mentioned factors with the risk of ANS. A total of 402 patients were first identified by the bivariate analysis, factors with p < 0.1 were further identified in the multivariate analysis. In the bivariate analysis, ANS was significantly more common in patients aged 51–60 years (OR,2.24; CI,1.15–4.39; p = 0.02) (as compared to age ≤ 30 years), non-Han ethnicity (OR,6.92; CI,1.42–33.78; p = 0.02) (as compared to Han ethnicity); pretreatment serum RPR titer 1:32 (OR,6.33; CI,1.87–21.40; p = 0.00), serum RPR titer 1:64 (OR,4.57; CI,1.19–17.6; p = 0.03) (as compared to ≤ 1:2), and ≥ 1:64 (OR,7.34; CI,1.96–27.43; p = 0.00) (as compared to 1:1). In the multivariate analysis, ANS was not significant in patients with pretreatment serum RPR titer 1:64 (p > 0.05). ANS remained significantly more common in patients aged 51–60 years (AOR,2.28; CI,1.11–4.66; p = 0.03) (as compared to age ≤ 30 years), non-Han ethnicity (AOR,9.11; CI,1.78–46.78; p = 0.01) (as compared to Han ethnicity), serum RPR titer 1:32 (AOR,5.12; CI,1.34–19.47; p = 0.02) and ≥ 1:64 (AOR,5.69; CI,1.34–24.20; p = 0.02) (as compared to 1:1). Patients aged 51–60 years, of other ethnicity, and with serum RPR titer 1:32 and ≥ 1:64 were 2.28-fold, 9.11-fold, 5.12-fold and 5.69-fold, respectively, more likely to have ANS. However, other factors, such as gender, marital status, fold decline of serum RPR titer after treatment, therapeutic regimen, time between treatment and lumbar puncture were not relevant factors for ANS.

## Discussion

Studies have documented the incidence of neurosyphilis in different stages of syphilis (primary, secondary, latent) or among various populations (HIV-negative, HIV-positive), and relevant factors of neurosyphilis among HIV-negative and/or HIV-positive syphilis patients^6,10,11^. However since ANS is difficult to diagnose^12^. its incidence and relevant factors remain largely unknown. Some studies had demonstrated that serofast state was common in syphilis with an incidence rate of 35.2–44.4%^13^. There was a certain association between ANS and serofast state, but the incidence of ANS among asymptomatic serofast syphilis patients in China requires further studies^9,10,14^. Therefore, we examined the incidence of ANS in asymptomatic syphilis patients with serofast state. Our results indicated that 139 of 402 patients had ANS, the incidence was 34.6%, higher than that of ANS among latent syphilis (13.5%) and syphilis with all stages (13.5%)^7^, which proved that ANS had a high incidence among serofast syphilis patients. About 35% of ANS patients developed symptomatic neurosyphilis^8^. Hence, ANS among serofast syphilis patients should be given more attention by clinicians. Of the 139 ANS patients, CSF WBC abnormality had the highest proportion of 82.7%, which was consistent with the highest proportion of CSF WBC abnormality in neurosyphilis patients with serofast state^10^. CSF WBC abnormality had high diagnostic value both in ANS and neurosyphilis. Our results were inconsistent with some studies on neurosyphilis in untreated early syphilis patients, which showed that CSF WBC abnormality accounted for the highest proportion among HIV-positive and total population, but protein abnormality had the highest proportion among HIV-negative patients^11^.

From the ROC curve, serum RPR titer 1:16 was the best cutoff value (sensitivity 32.4%, specificity 87.8%) for the diagnosis of ANS. Serofast syphilis patients with a serum RPR titer ≥1:16 had high risk of ANS. Some researches used a ≥4-fold decline as serological cure criteria^15,16^, but in our study, patients with a 4-fold decline of serum RPR titer after treatment who became serofast for more than one year showed similar chances of developing ANS, which was inappropriate as serological cure criteria.

Many studies showed that HIV-positive syphilis patients were more likely to be complicated by neurosyphilis, especially ANS^17^. Neurosyphilis in HIV-positive patients was related to CD4 ≤ 350 cells/ml, serum RPR titer ≥ 1:32, male gender, and an HIV viral load (VL) above 10000 copies/mL^12,17–19^. Recent researches showed CSF CXCL13 >10 pg/ml as a predictor of neurosyphilis^20^. Our results indicated that the patients with serum RPR titer 1:32 and ≥1:64 had 5.12-fold and 5.69-fold increased risk of ANS, respectively; and patients aged 51–60 years had a 2.28-fold increased risk of ANS. Furthermore, 60.9% of these patients had ≥2 years syphilis history between initial treatment and lumbar puncture. Unlike neurosyphilis, male patients did not have a high incidence of ANS in our study. Although the incidence of ANS in males (39.4%) was higher than in females (31.9%), it was not statistically significant.

This study had several limitations. Prognosis, which was obviously influenced by the presence and extent of CSF abnormalities^7^, could not be observed in our study. Also, other ethnicity patients were 9.11-fold more likely to develop ANS, but there were only nine other ethnicity patients, and more patients are needed to confirm this finding. Further investigations of ANS in different stages and HIV-positive serofast syphilis patients are essential. Patients >51–60 years with serum RPR titer ≥1:16 are more likely to have ANS. These patients should be recommended to undergo lumbar puncture and neurosyphilis treatment to prevent them from developing symptomatic neurosyphilis. Our findings can be used to guide clinicians in the identification of ANS among syphilis patients with serofast state.

## Methods

### Study Design and Ethics Statement

All patients in this study were enrolled from the clinical database of Peking University Ditan Teaching Hospital between September 2008 and August 2016. According to the 2008 European Guidelines on the Management of Syphilis and CDC guidelines in USA^21,22^, All of the enrolled patients were HIV-negative, and both of serological TPPA and RPR positive at any stage of syphilis; Serofast status was defined as (1) in the early syphilis patients who have been at least six months after initial recommended treatment and regular follow-up, a 4-fold decline in serum RPR titer was not observed even after additional treatment; (2) RPR titer was less than 4-fold decline more than one year after treatment in late latent syphilis; and (3) those who had 4-fold decline of serum RPR titer after initial treatment, but became no seroreversion and any decline in nontreponemal antibody titers for more than one year^13,15,21^. All patients underwent lumbar puncture and CSF tests for ANS at the Peking University Ditan Teaching Hospital. Patients with HIV-infection, reinfection of syphilis, symptomatic neurosyphilis, or other neurological diseases were excluded. Asymptomatic neurosyphilis is defined by the presence of one or more CSF abnormalities (pleocytosis, elevated protein concentration, or reactive CSF Rapid plasma regain card test [CSF RPR] test) in persons with serologic evidence for syphilis but no neurologic signs or symptoms^3,22,23^. ANS is characterized by reactive CSF RPR, or negative CSF RPR but CSF WBC count >5 × 10 6/L, or negative CSF RPR, CSF WBC count ≤5 × 10 6/L but CSF protein concentration >45 mg/dL, and without any neurological signs and symptoms. The other neurological diseases were excluded based on clinical history and physical examination. According to the diagnostic criteria, the patients were divided into two groups: the ANS group, which met the ANS criteria listed above, and the serofast syphilis group (excluding neurosyphilis). All methods were carried out in accordance with the relevant guidelines and regulations. Written informed consent was obtained from all participants, This study was approved by the Institutional Ethics Committee of Peking University Ditan Teaching Hospital.

### Laboratory methods

Laboratory analyses, including RPR (Shanghai Rongsheng Biological Pharmaceutical Co., Ltd, China) and Treponema pallidum particle agglutination (TPPA) (Fujirebio, Tokyo, Japan), were conducted as per the manufacturers’ instructions.

### Analysis and Statistics

Data was analyzed by IBM SPSS version 19.0. Figures were drawn using GraphPad Prism 5. Continuous variables were described using median and interquartile range (IQR), while categorical variables were described by numbers and percentages. Associations between categorical variables were assessed by the chi-square test. The ROC curve of the serum RPR titer was drawn and the best cutoff was found. Bivariate analysis was utilized to determine the factors associated with ANS. The odds ratios (OR) was estimated with 95% confidence intervals (CIs) from the bivariate analysis, and factors with p < 0.1 were further identified in the multivariate analysis. Adjusted odds ratios (AOR) with 95% CIs were also estimated from the logistic regression analysis. All hypotheses testing was two-sided, and p < 0.05 was considered to be statistical difference.
